# Evolución e impacto de la infodemia en la población infantil en tiempos de COVID-19

**DOI:** 10.26633/RPSP.2021.38

**Published:** 2021-05-12

**Authors:** Doménica Cevallos-Robalino, Nicolás Reyes-Morales, Mario Rubio-Neira

**Affiliations:** 1 Área de Salud, Epidemiología y Salud Colectiva, Universidad Andina Simón Bolívar Quito Ecuador Área de Salud, Epidemiología y Salud Colectiva, Universidad Andina Simón Bolívar, Quito, Ecuador.; 2 Psicología Clínica, Pontificia Universidad Católica del Ecuador Psicología Clínica, Pontificia Universidad Católica del Ecuador.; 3 Cardiología pediátrica, Hospital de Niños Baca Ortiz Quito Ecuador Cardiología pediátrica, Hospital de Niños Baca Ortiz de Quito, Ecuador.

**Keywords:** Infecciones por coronavirus, salud del niño, vulnerabilidad social, salud mental, comunicación, Coronavirus infections, child health, social vulnerability, mental health, communication, Infecções por coronavirus, saúde da criança, vulnerabilidade social, saúde mental, comunicação

## Abstract

La provisión de información oportuna, clara y correcta constituye una importante estrategia de control del pánico y de contención de un brote pandémico; sin embargo, al no ser una de las tareas prioritarias, da lugar a otro de los enemigos letales, que hoy en día enmarca otra crisis dentro de la pandemia por SARS-CoV-2 como lo es la infodemia, cuyas consecuencias han afectado a toda la población a nivel mundial, vulnerando especialmente a un grupo del que poco se habla, y que constituye nuestra población de estudio, los niños.

En este artículo se propone un análisis reflexivo que desmitifique falsos constructos acerca del bajo impacto de la pandemia por COVID-19, a través de una descripción cronológica de los diferentes eventos alrededor de la población infantil, que fueron construyendo los pilares de la infodemia en esta población, planteando tres momentos: el primero, al inicio de la pandemia, con una invisibilización de los niños ante el virus; el segundo momento de estigma bajo la figura de “super contagiadores” y el tercer momento donde se evidencia la crisis consecuencia del fracaso en la comunicación de información en este grupo poblacional.

El mundo se enfrenta a la pandemia y además a la necesidad actual de una justicia comunicativa, que incluya a los niños como grupo primario de atención. Con un abordaje desde la determinación social, se plantea una nueva normalidad que incluya el empoderamiento de los niños con información real y clara para combatir, desde su corta edad, el virus de la infodemia.

## Abstract (en)

The provision of timely, clear, correct information is an important strategy for controlling panic and containing a pandemic outbreak. However, as this task has not been prioritized in the context of the SARS-CoV-2 pandemic, a new lethal enemy has emerged that now poses another crisis, namely, the "infodemic", with consequences that have affected the entire population worldwide. In particular, it has increased the vulnerability of a group that is not often discussed: children, who constitute our study population.

This article provides an analysis aimed at demystifying false constructs about the low impact of the COVID-19 pandemic on the child population. It gives a chronological description of the different events that were the building blocks of the infodemic, affecting this population in three phases: first, at the onset of the pandemic, when its effects on children were ignored altogether; second, when children were stigmatized as "super-spreaders"; and third, when the crisis resulting from a failure to transmit information to this population group became evident.

The world is facing both the pandemic and a pressing need for communicative justice, which includes children as a primary target group. Taking a social determinants approach, this article proposes a new normal that includes using accurate and clear information to empower children to combat the infodemic virus from an early age.

## RESUMO

Fornecer informações claras, corretas e oportunas constitui uma importante estratégia para controlar o pânico e conter uma pandemia. Porém, por não ser prioritário, dá origem a mais um inimigo mortal que atualmente configura outra crise dentro da pandemia de SARS-CoV-2, a infodemia. A infodemia tem consequências que repercutem em toda a população mundial, prejudicando sobretudo um grupo pouco falado e que é a nossa população de estudo: as crianças.

Este artigo propõe uma análise reflexiva para desmitificar falsos constructos sobre o baixo impacto da pandemia de COVID-19, com a descrição cronológica dos diferentes eventos ocorridos que foram edificando os pilares da infodemia na população infantil. São considerados três momentos: o primeiro, no início da pandemia, com a invisibilização das crianças diante do vírus; o segundo, a estigmatização das crianças como “supertransmissores”; e o terceiro momento em que se evidencia a crise decorrente da falha em comunicar informações sobre este grupo populacional.

O mundo enfrenta a pandemia e também uma necessidade atual de justiça comunicativa que englobe as crianças como grupo primário de atenção. Com uma abordagem de uma perspectiva de determinação social, propõe-se uma nova normalidade que inclua o empoderamento infantil com a divulgação de fatos claros para combater, desde a tenra idade, o vírus da infodemia.

En Diciembre del 2019, surgió el nuevo Coronavirus, SARS- COV-2 en China, dando un giro a la historia mundial convirtiéndose en un brote pandémico, que ha provocado 112 521 610 casos y 2 495 881muertes hasta finales de Febrero del 2021 (1), afectando a todos los países a nivel mundial. Los estudios realizados en las pandemias pasadas de SARS y MERS, determinaron que, durante la primera fase, con la finalidad de disminuir la transmisión, la incidencia de casos y la tasa de mortalidad fue imprescindible la implementación de políticas de salud pública como cuarentenas domiciliarias, aislamiento de personas vulnerables y distanciamiento social (2,3); y además, una estrategia vital que demostró prevenir el pánico y contener el brote, que consistía en la provisión de información oportuna, clara y correcta. Al no ser una de las tareas prioritarias, se ha dado lugar a uno los enemigos más letales, que hoy en día enmarca otra crisis dentro de la pandemia por SARS-COV-2, la llamada infodemia o sobreabundancia en el volumen de la información real y falsa, entre rumores, teorías conspirativas, discursos estigmatizantes, y productos pseudocientíficos (4), cuyas consecuencias han afectado a toda la población a nivel mundial, vulnerando especialmente a un grupo del que poco se habla, y que constituye nuestra población de estudio y análisis, los niños.

La Convención por los Derechos del Niño (CDN) establece que todo niño, niña y adolescente tiene derecho a recibir información que sea adecuada para su edad, y al mismo tiempo, debe ser protegido de cualquier información que sea perjudicial para su desarrollo (5). En situaciones de desastre o crisis, son los adultos que viven con ellos los responsables de ayudarles a conocer la realidad, brindando la información real y apropiada para su edad (6).

Tradicionalmente los medios de comunicación son proveedores de información; sin embargo, también figuran como actores principales en la formación de valores, creencias y opiniones acerca del mundo externo, dependiendo del espacio, cultura y modos de vida de cada población (7). En el contexto de una crisis de salud pública global, los medios de comunicación cumplen un rol vital, que no se condiciona a la entrega de información verídica, sino que incluso puede contribuir a la contención del brote (8) para lo cual, las noticias son productos importantes (7).

Los espacios de convivencia entre los niños y los adultos se han ido transformando a través del tiempo. En la actualidad, por ejemplo, la televisión o la radio son considerados un actor más dentro del entorno social y familiar, incluso en medios con bajos ingresos económicos (9), siendo la televisión el centro de atención y motivación para las reuniones y momentos familiares. Por lo tanto, si actualmente las noticias son el producto de mayor consumo, surgen las preguntas: ¿cómo manejar la información en esta población?, ¿a qué están siendo expuestos los niños?, ¿deben conocer la realidad y dimensión de la pandemia? ¿cómo abordar con ellos la muerte y otros temas para no generar estrés, miedo o trauma? Esta problemática será abordada en este artículo con el fin de visibilizar cronológicamente el impacto de la infodemia alrededor del COVID-19 sobre el desarrollo de los niños, para lo cual se propone un análisis reflexivo que desmitifique falsos constructos acerca del bajo impacto de la pandemia en esta población y permita visibilizar la realidad de la pandemia en los niños.

## DESARROLLO Y ANÁLISIS

**FIGURA 1.**
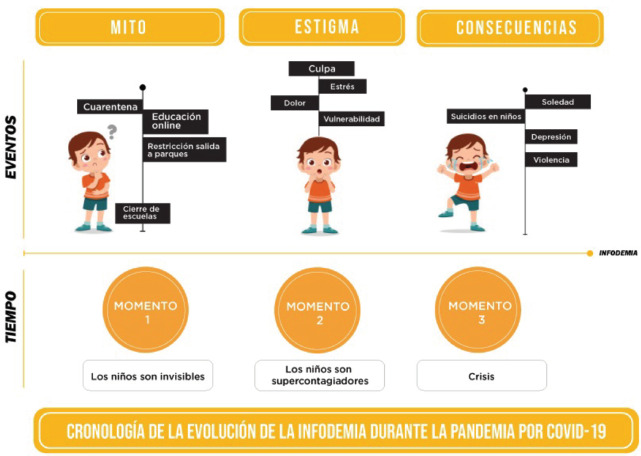
Cronología de la evolución de la infodemia durante la pandemia por COVID-19

El patrón de consumo de medios de comunicación durante la pandemia se ha incrementado exponencialmente, a través de medios tradicionales y digitales, como en el caso de Estados Unidos donde se reportan aumentos de consumo de noticias del 62%, llamando particularmente la atención el notable aumento de consumo en población joven, del 32 al 86% (10); y en países Latinoamericanos, donde se reportan ingresos a sitios web de noticias hasta en un 80% (7). Los estudios afirman que la exposición constante a noticias acerca de la crisis, sus impactos, y riesgos, vulneran aún más a la población, incrementando sus sensaciones de victimización, incertidumbre y dolor, alterando el proceso normal de recepción de información (7). La presente discusión se encuentra organizada en una serie temporal planteado en tres momentos que describen cómo se originó el virus de la infodemia en la población infantil: Momento 1 (M1) al inicio de la pandemia (cuando los niños “eran invisibles al impacto por COVID-19”); Momento 2 (M2) cuando se les catalogó como supercontagiadores; y el Momento 3 (M3) o de crisis donde se evidencian las consecuencias del fallo en la comunicación de información en este grupo poblacional (figura 1).

**Elaboración:** Cevallos D.

## LÍNEA DEL TIEMPO DE LA CONSTRUCCIÓN DE LA INFODEMIA EN LA POBLACIÓN INFANTIL.

### Momento 1. El virus no afecta a los niños. El mito.

En plena primera fase de la pandemia, en enero del 2020; los estudios de investigación describían en ese momento la poca o nula probabilidad en los niños de desarrollar infección grave (11); sin embargo, en el caso de presentarse, no desarrollaban manifestaciones clínicas, siendo la mayoría, asintomáticos. Estos datos se confirmaban con los reportes epidemiológicos publicados con cifras del 1.7% de casos de COVID-19 en la población infantil en países como Estados Unidos, y en menos del 2% en otros países como España e Italia (12). En contexto de esta baja incidencia clínica de COVID-19 en los niños, se generó socio-estructuralmente un mito que comenzó a difundirse: “COVID-19 no afecta a la población infantil”, constituyendo uno de los falsos pero sólidos pilares de la infodemia en esta población, que invisibilizó las problemáticas alrededor del devastador efecto de la pandemia en todos los aspectos integrales del desarrollo infantil, ya que mientras se difundía la información de que a los niños no les afecta la pandemia, la realidad a nivel mundial fue marcada por la interrupción en los sistemas de protección infantil: el cierre de escuelas en 107 países, lo cual afectó a más de 860 millones de niños (13) provocando de forma subsecuente otros problemas. Apareció la inseguridad alimentaria, ya que se perdieron aportes nutricionales diarios en un alto porcentaje de la población infantil que recibía alimentación en sus establecimientos educativos; la “normalidad” entendida como la vida cotidiana en familia, rutinas establecidas, interacción social y recreación con actividades deportivas, se vio alterada con un aumento de hábitos malsanos como el sedentarismo y el tiempo frente a pantallas, entre otros efectos (12). Las capacidades de los padres y madres para sostener a sus hijos en cuestión tiempo y economía fueron sobrepasadas, perdiéndose la productividad parental, ya que algunos, obligados a salir a trabajar tuvieron que dejar a sus hijos a cargo de sus abuelos, vecinos o en su defecto, solos; dando lugar a casos de negligencia. De igual manera, en diversos países se reportaron incrementos de casos de violencia física, psicológica y sexual en contra de niños, niñas y adolescentes, además de la falta de acceso a instituciones de protección y justicia. Asimismo, se limitó su acceso a servicios de atención médica; y se limitaron sus actividades de recreación con restricción de salida a los parques. Los niños tendrían un gran reto para adaptarse a estas nuevas condiciones que se impusieron de forma brusca y sin aviso (12).

Algunas decisiones tomadas alrededor de todos los acontecimientos durante el brote pandémico son debatidas hasta el día de hoy, en términos de su eficacia en la disminución de la transmisibilidad y por el eterno dilema acerca de si es mayor el beneficio que el riesgo en la salud integral infantil, siendo un claro ejemplo el cierre de escuelas, que tuvo su base en evidencia científica reportada en pandemias pasadas como de la influenza, donde los niños eran la principal fuente de contagio siendo los más susceptibles y sintomáticos en aquella infección (14). Hasta el día de hoy, no se tiene certeza si la decisión del cierre masivo de escuelas ha resultado un beneficio más que un perjuicio a los niños, en el contexto de una especie de amenaza al derecho de la educación versus el derecho a la salud.

### Momento 2. Los niños son los supercontagiadores. El estigma.

La evolución cronológica de la problemática de la infodemia en los niños continúa con un aparente cambio en el comportamiento epidemiológico de la transmisión. En términos del manejo de la información, la divulgación pasó de ser un mito al estigma, otra de las categorías comunes de la infodemia (15), que señalaba a los niños como los “super spreaders” del virus (11,16). Los niños pasaron de ser invisibles a ser los supercontagiadores asintomáticos. Este es el proceso de transición que daría lugar al Momento 3, de crisis, donde los niños al sentirse señalados y casi culpables del contagio de sus familiares y amigos, empezaron a evidenciar conductas desadaptativas que reflejaban el poco soporte para su resiliencia y comprensión de la realidad. (17)

### Momento 3. La crisis. Consecuencias e impactos.

Latinoamérica ha sido una de las regiones más afectadas por la pandemia, especialmente en Brasil, Perú y Ecuador, países donde el manejo de la crisis ha sido muy cuestionado (18). Este último país constituye caso de estudio, siendo uno de los países con más casos confirmados en la región, en donde particularmente llama la atención la incidencia de trastornos mentales en los niños y adolescentes que se incrementó exponencialmente durante la pandemia. Los reportes de años pasados mostraban que el 63,5 % de la población no presentaba signo alguno de depresión; sin embargo, durante el periodo de confinamiento en la pandemia, se estima que casi el 50 % de la población presenta actualmente síntomas de depresión (12). Otro estudio (19) describió que el 38,5% de un grupo de más de 3 000 niños, niñas y adolescentes, se han sentido angustiados y el 24,5% deprimidos; durante esta pandemia. Las principales preocupaciones identificadas en los niños y adolescentes son el temor a que las personas de su familia enfermen o mueran por COVID-19 (90,1%); seguido del temor a que se acaben los recursos para comprar comida (68,8%); el temor a que los miembros de su familia pierdan su trabajo (44,3%); a que no puedan aprobar el año escolar (27,3%), entre otros. Acerca de la comunicación intrafamiliar y con quién hablan sobre su estado de ánimo, las respuestas fueron: con mamá (61,1%), con papá (29,5%), con hermanos (25,7%), amigos (21, 8%) o con nadie (19,1%) (19). Esta crisis se enmarca además por la adaptación forzada a la tecnología que los niños de todas las edades han tenido que atravesar, siendo el Internet, el actual recurso básico para la educación y para la recreación, que en conjunto, ha ubicado a los niños como blanco de los ciberacosadores, dejándolos vulnerables a información sin filtro que ataca su adecuada concepción de la realidad, y los expone de frente a la violencia, vulneración de derechos de privacidad, derechos sexuales, y derechos a la información (5).

**FIGURA 2.**
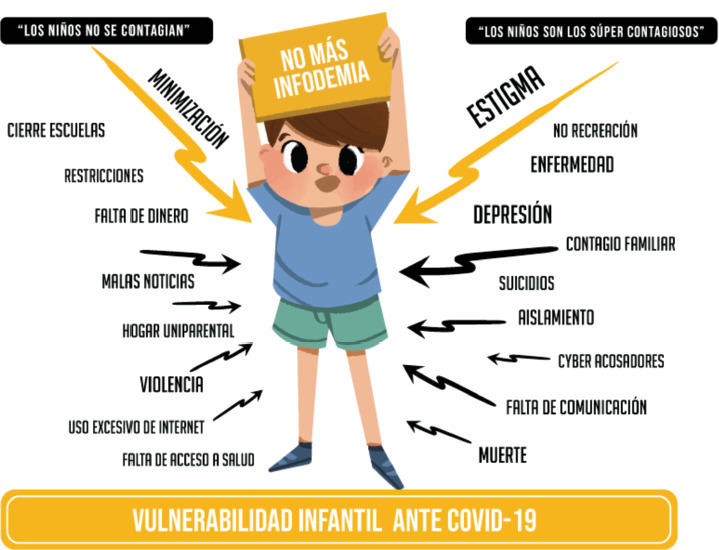
Vulnerabilidad Infantil ante COVID-19

Las consecuencias en la población infantil han sido diversas a nivel global; sin embargo, el mayor impacto descrito es el psicoemocional (19). La exposición prolongada a los desastres naturales o eventos como la actual pandemia, constituyen un choque, en la línea de vida de cualquier individuo (14,20), principalmente en los niños (figura 2). Este choque se incrementa cuando la información no es adecuadamente transmitida, generando altos niveles de estrés y dando lugar, posteriormente a trastornos o enfermedades orgánicas; efecto específicamente demostrado en niños intraútero o preescolares, que tienen un sistema nervioso altamente sensitivo a la adversidad (20). Este es el fundamento de las llamadas “experiencias infantiles adversas” y su efecto en la salud física, mental y emocional en la adultez; por lo tanto, el abordaje de una pandemia y de la infodemia como una experiencia infantil adversa, se vuelve imprescindible, a fin de generar estrategias que consideren este aumento de potenciales adversidades, a través de programas de soporte y sistemas de resiliencia, como el implementado antes de la pandemia, en el estado de California, en Estados Unidos, donde a través de una investigación universal de adversidad en los niños, se logra establecer procesos de vulnerabilidad (21), útil en términos de salud pública infantil para prevención de enfermedades.

**Elaboración:** Cevallos D.

En conclusión, el presente documento analizó la evolución e impacto de la infodemia sobre la población infantil en el contexto de la pandemia por COVID-19. A través de un análisis en serie, del tiempo de la evolución de la pandemia, lográndose identificar tres momentos que estructuraron la infodemia, incluidos los mitos y estigmas relacionados con la población infantil: primer momento de invisibilización de los niños ante los impactos de la pandemia del COVID-19; un segundo momento de estigma bajo la figura de “supercontagiadores” del virus y un tercer momento de consolidación de la infodemia sobre la población infantil consecuencia del fracaso de los mecanismos de comunicación adecuados para su edad.

De tal manera, con base a la información recopilada y el análisis realizado, se concluye que la población infantil ha sido afectada en diversas dimensiones que amenazan su desarrollo integral, siendo afectadas distintas esferas del desarrollo infantil como son: protección; educación; salud física y mental; alimentación y nutrición; condiciones de vida, entre otros.

Pocos gobiernos a nivel mundial han asumido la responsabilidad de cubrir la necesidad de la población infantil de tener acceso a la información veraz y adecuada para su edad; lo cual, finalmente se suma a la falta de evidencia científica y de lectura crítica de los estudios, que han dado lugar a la consolidación de asociaciones erróneas entre la enfermedad y otros procesos en los niños, desencadenando pánico de forma masiva e incluso actitudes estigmatizantes que terminan en conductas deshumanizantes para quienes han contraído el virus, fenómenos que han ocurrido aún entre los niños y niñas.

Frente a la información analizada, la principal recomendación se basa en la elaboración de estrategias y acciones específicas consolidando un sistema de comunicación eficaz, que permita combatir la infodemia y fortalecer los mecanismos de protección integral de la población infantil.

Por otra parte, se recomienda que los gobiernos establezcan alianzas estratégicas a través de sus instituciones de bienestar social, educación y salud, con medios de comunicación nacionales y locales; públicos, privados y comunitarios: radio, televisión, prensa escrita, redes sociales, comunicadores comunitarios, *influencers* digitales, entre otros, para difundir contenidos acordes a la edad de los niños, como recursos animados y didácticos en forma de cuentos o caricaturas; y además fomentar programas de atención psicológica especializada para facilitar la resiliencia infantil y aliviar el sufrimiento psicológico. En el contexto latinoamericano, se recomienda integrar la información en lenguas nativas de las diversos pueblos y comunidades, que permitan un abordaje intercultural de la crisis.

De forma simultánea, los programas de salud mental deben fortalecerse, trabajando sobre la importancia de la expresión de las emocione de los niños, asegurando la adecuada comunicación comunitaria y familiar. Además, se debe fomentar el uso seguro del Internet, el control parental de acuerdo a la edad, la protección de los datos e identidad de los niños y niñas, programas de enseñanza de verificación y análisis crítico de la información que receptan los niños. Se propone la inclusión de programas de educación emocional en la malla curricular educativa actual que permita gestionar las emociones de los niños, además del seguimiento por personal de educación y bienestar social según el diagnóstico familiar para detectar y abordar tempranamente casos de violencia o negligencia, y evitar consecuencias devastadoras como suicidios u homicidios.

La necesidad actual radica en una justicia comunicativa, que incluya a los niños como grupo primario de atención. El manejo de la información desde la determinación social es vital, donde se entiende que la información termina siendo un bien de consumo que tiene un precio y responde a intereses de ciertos grupos, presentando coberturas sensacionalistas y fatalistas que inducen al pánico y omiten el cuidado de la salud mental del consumidor. El llamado es a los tomadores de decisiones a la creación de políticas públicas que regulen contenidos y resguarden la salud de los niños. La crisis desencadenada por la pandemia abre camino a la oportunidad de generar nuevas políticas que beneficien integralmente a la niñez, creando una nueva normalidad que incluya el empoderamiento de los niños con información real y clara para combatir, desde su corta edad, el virus de la infodemia.

## Declaración.

Las opiniones expresadas en este manuscrito son responsabilidad del autor y no reflejan necesariamente los criterios ni la política de la *RPSP/PAJPH* y/o de la OPS
